# Analysis of Environmental Conditions Effect in the Phytochemical Composition of Potato (*Solanum tuberosum*) Cultivars

**DOI:** 10.3390/plants9070815

**Published:** 2020-06-29

**Authors:** Iván Samaniego, Susana Espin, Xavier Cuesta, Verónica Arias, Armando Rubio, Wilma Llerena, Ignacio Angós, Wilman Carrillo

**Affiliations:** 1 Department of Nutrition and Quality, National Institute of Agricultural Research (INIAP), Panamericana Sur Km. 1, Mejia 170516, Ecuador; ivan.samaniego@iniap.gob.ec (I.S.); susana_espin@hotmail.com (S.E.); xavier.cuesta@iniap.gob.ec (X.C.); veronica.arias@iniap.gob.ec (V.A.); arubioc@yahoo.es (A.R.); 2 Facultad de Ciencias Pecuarias, Ingeniería en Alimentos, Universidad Técnica Estatal de Quevedo, Km 7 1/2 vía Quevedo-El Empalme, Los Ríos 120313, Ecuador; wm.llerena@uta.edu.ec; 3 Departamento de Agronomía, Biotecnología y Alimentación, Edificio Los Olivos, Campus Arrosadia, Universidad Pública de Navarra (UPNA), Pamplona 31006, Espana; ignacio.angos@unavarra.es; 4 Department of Research, Universidad Técnica de Babahoyo, Av. Universitaria Km 21/2 Av. Montalvo., Babahoyo 120301, Ecuador

**Keywords:** potato, Solanum tuberosum, genotype, environments, antioxidant capacity

## Abstract

Crop productivity and food quality are affected by environmental conditions. The objective of this work was to evaluate the effect of the environment on the concentration of phytochemical components in several potato (*Solanum tuberosum*) cultivars. The content of vitamin C (ascorbic acid, AA), the total carotenoids content (TCC), the total polyphenols content (TPC), and the total anthocyanins content (TAC) of 11 potatoes varieties grown in Ecuador (Cutuglahua, Pujilí, and Pilahuín) was measured by the spectrophotometric method. The antioxidant capacity (AC) of potato cultivars was evaluated by the ABTS method. The AA concentration ranged between 12.67 to 39.49 mg/100g fresh weight (FW), the TCC ranged between 50.00 and 1043.50 μg/100g FW, the TPC ranged between 0.41 and 3.25 g of gallic acid equivalents (GAE)/kg dry weight (DW), the TAC ranged between 2.74 and 172.53 μg/g FW and finally the AC ranged between 36.80 and 789.19 μg of trolox equivalents (TE)/g FW. Genotypes (G), location (L), and interaction (G x L) were significant at *p* < 0.01. The genotype (G) showed a greater variation in the phytochemical contents. AA and TPC showed the highest correlation with the AC. A selection of genotypes with these characteristics can be used to develop germplasms with a high AC.

## 1. Introduction

Potato (*Solanum tuberosum* L.) is the third most important crop worldwide after wheat and rice, with 368,168,914 million tons of fresh weight of tubers produced in 17.60 million hectares during 2018 [1]. This tuber produces more dry matter and protein per hectare when compared to the main cereals [2]. In Ecuador, potatoes are one of the main crops in the Sierra region. In 2018, more than 22,099 hectares were harvested with a total production of 269,201 tons. This production represents an average yield of 12.18 t/ha [1] involving more than 82,000 producers. The production is mainly oriented towards the domestic market, where approximately 81.00% is marketed for fresh consumption and the rest is used by the processing industry to make chips or strips [3].

Potato cultivation in Ecuador takes place in the highlands, between 2700 and 3400 m altitudes in the ten Sierra provinces. The most representative areas by the volume of production are the North: Carchi and Pichincha; the Center: Cotopaxi, Tungurahua, Bolívar, and Chimborazo, and finally the South, with Cañar and Azuay. Most potato producers (76.00%) are small producers with production units of less than 5 ha, mostly located in marginal areas, with low production.

Potato is rich in carbohydrates, with significant amounts of protein and a good balance of amino acids. The potato tuber has a high dietary fiber content when consumed with peel. In addition, it is rich in antioxidant components. The main antioxidants are phenolic compounds, vitamin C, flavonoids, and carotenoids [4]. Potatoes have four types of polyphenols components: phenolic acids, flavonols, flavan-3-ols, and anthocyanin components. The most abundant polyphenol in potato is the chlorogenic acid, representing around 90.00% of total polyphenols content (TPC). Crypto-chlorogenic, neochlorogenic, and caffeic acids are found in lower concentrations than chlorogenic acid in the different potato cultivars [5,6]. Gumul et al. (2011) described five potatoes cultivars from Poland with TPCs between 2.12–3.27 mg catechin/g, dry weight (DW), the total flavonoids content (TFC) with values of 0.71–1.00 mg rutin/g DW, and flavonols content with values of 0.01 to 0.27 mg quercetin/g DW. Potato varieties presented antioxidant activity measured by Ferric-reducing antioxidant power assay (FRAP) and 2,2-azinobis,3-ethyl-benzothiazoline-6-sulfonic acid (ABTS) methods with values of 6.20–14.35 mM Fe/kg DW and 18.37–28.70 mM Trolox/kg DW.

Antioxidant activity relates to TPC [7]. Deußer et al. 2012 described 16 potato cultivars field-grown in Luxembourg with TPC values ranging from 0.40 mg GAE/g DW to 5.40 mg GAE/g, DW in the potato flesh [8]. Al-Weshahy and Rao, (2009) described six potato cultivars grown in Canada with TPC values ranging from 1.51 to 3.33 mg GAE/g of potato peel. These varieties presented an antioxidant activity measured by FRAP with values of 0.28–0.55 mM TE/g in the potato peel extract and free radical scavenging activity (FRSA) with values ranging from 82.00% to 90.00% FRSA. [9].

Potato is an important source of compounds with antioxidant capacity. Several studies showed that potatoes are the third food source, after oranges and apples in the daily intake of antioxidant components due to its high daily consumption in the diet [10]. The positive effect of antioxidant components on human health has been reported by several authors [11,12,13,14,15]. These studies show the possibilities of enhancing the quality of diets when consuming food with high antioxidant properties. Thus, there is a continuous search for new natural compounds with antioxidant capacity such as proteins, hydrolysates, peptides, polyphenols, carotenoids, and flavonoids components [16,17,18,19,20]. Many epidemiological studies relate the prevention of cardiovascular diseases with the consumption of polyphenols in the diet. Polyphenols are described to prevent oxidative stress, cell damage, DNA, protein damage, and lipid peroxidation processes. Its consumption is also related to the prevention of neurodegenerative diseases such as Alzheimer’s and Parkinson’s [21,22,23,24].

The biodiversity of species and subspecies plays an important role for food security and human nutrition because different varieties of the same species may contain different content of nutrients and bio-compounds. It is therefore important to generate information on the nutrient and bio-compounds content [25]. These studies can motivate the conservation of these regional crops and encourage consumer interest in these potato cultivars.

The concentration of the phytochemicals present in the potato tuber can be affected by several factors such as genotype, environment, post-harvest, and processing conditions. Hamouz et al. (2011) studied the effect of the environment and fertilization conditions on TPC of nine potato cultivars grown in four sites in the Czech Republic. They found that environmental conditions as drought and high altitude affect TPC in potato varieties [26].

The aim of this study was to determine the effect of the environment on the concentration of antioxidant compounds: total carotenoids content (TCC), TPC, total anthocyanins content (TAC), ascorbic acid (AA), and its correlation with the antioxidant capacity (AC) in eleven potato cultivars. These domesticated varieties were selected from previous studies for their quality characteristics and their presence in the Ecuadorian markets.

## 2. Materials and Methods

### 2.1. Plant Material

Eleven potato cultivars were used: *Red chaucha, Yellow chaucha, Natividad, Black coneja, Yana shungo* (“Black heart”)*, Puca shungo* (“Purple heart”)*, Victoria, Libertad, Fripapa, Puña,* and *Uvilla.* All potato cultivars were tested in three zones of the Ecuadorian Andean region: zone 1, Cutuglahua, Experimental Station Santa Catalina of National Institute of Agricultural Research (INIAP) (3058 m above sea level, masl); zone 2, Pujilí, Province of Cotopaxi (3098 masl); and zone 3, Pilahuín, Province of Tungurahua (3489 masl). The tuber samples were taken from plots located in a randomized complete block designed with three replications in three locations. Each treatment consisted of a plot of 4 rows. Each plant was planted at 0.30 m distance between plants and 1.10 m distance between rows. Potato seeds from the INIAP germplasm bank were used.

Once tubers reached commercial maturity, they were harvested 160 days after sowing in Santa Catalina, 140 days in Pujilí, and 130 days in Pilahuín. A sample of 2 kg of tubers free from damage and diseases was taken from each treatment. The samples were stored in a paper sleeve, labeled, and sent to the Nutrition and Quality Department of INIAP for their respective analysis.

### 2.2. Preparation of the Sample

From each treatment, 4 to 5 representative tubers were taken, washed with abundant water to remove the residues from the soil, and cut into 4 longitudinal sections. Moreover, 3 to 5 slices were obtained from each section and cut into small squares. The sample was divided into two parts, the first part was used for the analysis of vitamin C (AA) and total carotenoids content (TAC). This first part was used fresh. The second part (500 g) was subject to a drying process by lyophilization using Lab Kits FD18 (Hong Kong, China) for 24 h, then grounded using a coffee mill to obtain a fine powder. This second part of the sample was used to analyze TPC, TAC, and AC.

### 2.3. Vitamin C (AA)

The ascorbic acid (AA) concentration was evaluated by the spectrophotometry method described by Burgos et al. (2009a) [27]. The 7.50 g samples were treated with a solution of oxalic acid and acetone 0.40 and 20.00%, respectively. Samples were mixed in an Ultra Turrax for 1 min at 4000 rpm. The extract was filtered using a vacuum through Whatman filter paper and taken to 50 mL with the same extraction solution. One milliliter of the extract reacted with 9 mL of 2.60 dichloroindophenol 1.60% (Hopking/Williams 1053/3) for 1 min. The absorbance was measured at 520 nm in a UV-VIS spectrophotometer (Shimadzu 2201, Shimadzu Corp., Kyoto, Japan). The AA concentration was quantified using a standard AA curve. The results were expressed as mg ascorbic acid AA/100 g of fresh weight (FW).

### 2.4. Total Carotenoids Content (TCC)

The TCC analysis was performed according to the method described by Llerena et al. (2012) [28]. The extraction was done with acetone. The carotenoids were transferred to petroleum ether after saponification, to release the esterified carotenoids. The samples were selected according to the color of the pulp, 6 to 8 g for samples with a pale-yellow flesh color; 4 to 5 g for samples with an intermediate yellow pulp color, and 2 to 3 g for samples with an intense yellow pulp. Samples were mixed with acetone in an Ultra Turrax Teckmar T10 homogenizer for 1 min at 4000 rpm. The extraction was repeated until the residue had no color. The extract was transferred to a 500 mL separation balloon with petroleum ether, then washed 3-4 times with water to remove any acetone residue. The extract was saponified with a 20.00% KOH solution. The resulting saponified extract was taken to a volume of 25 mL with petroleum ether. The absorbance was measured at 450 nm. The extinction coefficient for TCC mixtures was 2592. TCC was expressed in μg β-carotene/100g FW.

### 2.5. Total Polyphenol Compound (TPC)

The TPC content was measured according to the method described by Andre et al. (2018) [29] with some modifications. The polyphenols were extracted with 70.00% methanol from lyophilized samples under constant stirring for 1 min, followed by ultrasound for 10 min. The extract was centrifuged, filtered, and calibrated to 50 mL with the extraction solution. An aliquot was taken and mixed with distilled water, Folin Ciocalteau reagent and 20.00% sodium carbonate. The absorbance was measured at 765 nm. The TPC quantification was performed using a standard calibration curve for gallic acid (GA). TPC was expressed in g gallic acid equivalents GAE/kg DW.

### 2.6. Total Anthocyanin Content (TAC)

The TAC analysis was performed using a technique adapted from the Jansen and Flamme (2006) method [30]. The anthocyanins were extracted with a solution of ethanol: 1.5 M HCl (80:20 *v:v*) by continuous stirring for one min, followed by ultrasound for 10 min, then centrifuged, filtered, and gauging the extracts at a volume of 25 mL. Quantification was performed by measuring the absorbance of 742 nm, using the molar extinction coefficient of malvidin-3-p-coumaryl-glucoside (3.02 × 10^4^). TAC was expressed in μg cyanidin-3-gluscoside chloride/100g FW.

### 2.7. Antioxidant Capacity (AC)

To measure AC, an extraction with a methanolic solution was carried out. The quantification was performed spectrophotometrically. The ABTS method was used to measure the antioxidant capacity (2,2’azinobis- (3-ethylbenzthiazolin 6-sulphonic acid) and a standard curve of an antioxidant 6-hydroxy-2,5,7,8-tetramethylchroman 2-carboxylic acid 2-carboxylic acid (Trolox) was used. The absorbances were measured at 734 nm. The AC was expressed as μg of Trolox equivalents TE/g FW [31].

### 2.8. Statistical Analysis

To measure the AA, TCC, TPC, TAC, AC variations an analysis of variance (ANOVA-one way) was performed for each site, considering a complete design at random with three repetitions. The averages were compared using the Tukey test at 5.00%. The effect of the environment (Cutuglahua, Pujilí and Pilahuín) and the genotype x location (G x L) interaction was analyzed through ANOVA, considering the genotypes as a fixed factor and the sites as randomized effects. Prior to the analysis of the variance, the data were subject to a normality Shapiro–Wilk test and transformed using square root. All statistical tests were performed using the 2010 INFOSTAT version.

## 3. Results and Discussion

### 3.1. Vitamin C (AA)

The AA concentration was determined in potatoes cultivars grown in the Cutuglahua, Pujilí and Pilahuín locations (Table 1). The analysis of variance (ANOVA) established significant differences (*p* < 0.01) for the AA concentration in each location. Potato cultivars presented AA values of 17.10 to 39.49 mg AA/100 g fresh weight (FW), (Cutuglahua) 13.05 to 27.59 mg AA/100 g FW (Pujilí) and 12.67 to 33.66 mg AA/100 g FW (Pilahuín). Previous studies reported AA content of potato cultivars grown in different countries. For example, native potato cultivars of the International Potato Center (CIP) had: AA values of 6.50 and 36.90 mg AA/100 g FW in [32]; AA values of 10.40 to 17.00 mg AA/100 g FW for potato cultivars from India [33]; AA values of 8.40 to 20.10 mg AA/100 g FW for Norwegian cultivars [34]; AA values of 8.80 to 24.10 mg AA/100 g FW for Canadian cultivars [35]; and AA values of 16.00 to 46.00 mg AA/100 g FW for Korean varieties [36]. The cultivar *Libertad* presented the highest AA values in the three locations tested with values between 33.66-39.49 mg AA/100 g FW, whereas the native cultivar *Puña* showed the lowest AA values between 12.67–20.81 mg AA/100 g FW. These results showed a great variation for the AA contents of the evaluated genotypes. The reported values were higher than those found in the previous studies, except for the Korean varieties. This probably happened because AA content analysis was performed in the central part of the tub.

### 3.2. Total Carotenoids Content (TCC)

Table 1 showed the results of quantification of TCC of potato cultivars grown in Ecuador. The ANOVA established significant differences (*p* < 0.01) for the genotypes studied. TCC concentration ranged from 87.70 to 1043.50 μg of β-carotene/100 g FW in Cutuglahua, 50.00 to 423.50 μg of β-carotene/100 g FW in Pujilí and 57.80 to 511.60 μg of β-carotene/100 g FW in Pilahuín. Calliope, Lobo and Sammán (2018) described the phytochemical composition of 25 genotypes of Andean potatoes grown in Argentina. They reported TCC values of 116.00 to 718.00 μg of β-carotene/100 g FW. The TCC values obtained in the Cutuglahua location were higher [25].

These values are lower compared to those reported by Burgos et al. (2009b) [32] who analyzed TCC in native potato cultivars finding values up to 1849.00 μg/100 g FW. However, the contents are higher compared to those reported by Breithaupt and Bamedi (2002) [37] who established TCC values between 58.00–175.00 μg/100 g FW in yellow flesh varieties and between 38.00 to 62.00 μg/100 g FW for white flesh varieties. Similarly, Brown (2005) [38] found a range of 35.00 to 795.00 μg/100 g FW in potato cultivars from USA.

The cultivars with the highest contents were *Red chaucha* with 423.50 - 1043.50 μg/100 g FW and *Yellow chaucha* with 341.20 to 423.50 μg/100 g FW. These varieties belong to the species group of *Solanum phureja*, already reported to have higher TCC contents compared to cultivars belonging to the *S. tuberosum* species group [30,39]. Burgos et al. (2012) [40] have reported the effect of boiling in total and individual carotenoids concentration of seven native Andean potato accessions 705,821 (*S. phureja*), 705,172 (*S. phureja*), two intermediate yellow fleshed: 704,393 (*Solanum goniocalix*), 701,862 (*S. goniocalix*) and three deep yellow fleshed: 702,472 (*S. goniocalix*), 705,799 (*S. phureja*) and 704,218 (*S. phureja*). They found values between 1233.00 to 7563.00 µg β-carotene/100 g DW in raw tuber and 1460.00 to 7648.00 µg β-carotene/100 g DW.

### 3.3. Total Phenolic Content (TPC)

Table 1 showed the content of TPC of potato varieties. The ANOVA established significant differences (*p* < 0.01) for the genotypes in each location. TPC had a range between 0.73 to 3.25 g GAE/kg DW from 0.41 to 1.77 g GAE/kg DW and from 0.57 to 1.44 g GAE/kg DW for Cutuglahua, Pujilí and Pilahuín, respectively. TPC values reported in this study are in accordance to the TPC values reported by Cuesta (2013) [41], in Ecuadorian native potato cultivars, with ranges of 0.94 and 4.08 g GAE/kg DW. These values are in line with those described by Lachman et al. (2008) [42] in European varieties with ranges between 2.46 and 4.81 g GAE/kg DW. These values are comparable to those reported by Andre et al. (2007) [11] in CIP potato accessions representing more than 60.00% of the potato collection variation. These authors found values with a range between 1.12 to 3.77 g GAE/kg DW. Only two accessions of skin and purple flesh reached values of 5.99 and 12.37 g GAE/kg DW, respectively.

The varieties *Yana shungo* (Kichwa name meaning “Black heart”) and *Puca shungo* (Kichwa name meaning “Purple heart”) had the highest TPC values in the localities of Cutuglahua and Pujilí with ranges of 1.87 to 3.25 g GAE/kg DW and of 1.65 and 1.77 g GAE/kg DW, respectively. Pilahuín varieties *Yellow chaucha* and *Red chaucha* had the highest contents with 1.44 and 1.29 g GAE/kg DW, respectively. Calliope, Lobo and Sammán (2018) analyzed TPC of 25 Andean potato cultivars. They found TPC values of 3.73 to 7.45 g GAE/kg DW. The TPC values of this study were lower than the values reported by Calliope, Lobo and Sammán, 2018. This situation indicates that different potato cultivars have different content of phytochemical components [25].

### 3.4. Total Anthocyanins Content (TAC)

Table 1 show that content of TAC of potato cultivars evaluated in the three locations presented great differences. The ANOVA established significant differences (*p* < 0.01) for genotypes within each location. The TAC range was as follows: between 3.28 and 107.43 μg of cy -3-glu/g FW, between 2.74 and 172.53 μg of cy-3-glu/g FW, and between 5.69 and 43.37 μg of cy-3-glu/g FW, for the localities of Cutuglahua, Pujilí, and Pilahuín respectively.

Potato cultivars with pigmented skin and flesh compared to non-pigmented potato cultivars showed the highest levels of anthocyanins. Thus, *Yana shungo* and *Puca shungo* cultivars showed the highest TAC contents in all locations with values between 34.67 and 172.53 μg of cy-3-glu/g FW. The variety *Black coneja* grown in Pilahuín presented TAC values of 43.37 μg of cy-3-glu/g FW, with skin of color between purple and blackish, and the presence of pigmentation in the flesh. Albishi et al. (2013) reported TAC of four potato cultivars from Canada. They found TAC values of 2.40 to 68.40 μg of cy-3-glu/g FW of potato peel extract and 0.07 to 6.40 μg of cy-3-glu/g FW of flesh potato extract. The TAC values of our study were higher than the ones reported in this study [43]. Brown et al. (2008) reported TAC values of 15.00 μg of cy-3-glu/g FW, in red fresh potato peel and 400.00 μg of cy-3-glu/g FW, in red potato peel [44]. Calliope, Lobo and Sammán (2018) described total monomeric anthocyanin (TMA) of 25 genotypes of Andean potatoes grown in Argentina. They found TMA values between 0.20 to 214.60 μg of cy-3-glu/g FW [25]. These values of anthocyanins were higher than those reported in this study.

### 3.5. Antioxidant Capacity (AC)

Table 2 showed the results of AC of potato cultivars. AC presented values between 228.75 to 789.19 μg of TE/g FW for Cutuglahua location, from 36.38 to 619.82 μg of TE/g FW for Pujilí location, and between 134.85 and 585.88 μg of TE/g FW for Pilahuín location. The analysis (one-way ANOVA) established differences between the varieties tested in each locality. Campos et al. (2006) [16] reported values of AC of 15 native potato cultivars with a range of 115.00 and 361.00 μg TE/g FW of AC. The results of our study were higher than the ones reported by Campos et al. (2006). Moreover, Reddivari et al. (2007) [45] evaluated AC of 25 selected cultivars from Texas. They found AC values of 47.00 and 783.00 μg TE/g FW. Victoria cultivar grown in Cutuglahua presented a higher value than that reported by Reddivari et al. (2007): the AC value obtained was of 789.19 μg of TE/g FW. Nemś et al. (2015) described AC of four cultivars of red and purple-fleshed potatoes from Czech Republic. They found AC values measured by ABTS of 0.64 to 9.01 µM TE/100 g DW of dried potato and 14.95 to 36.10 µM TE/100 g DW of potato flour [46]. Potatoes are used to the production of different foods processed as snacks. Nemś and Pęksa, (2018) reported the use of three purple-flesh cultivars to produce snacks. AC from these snack potatoes was evaluated using the ABTS method and found values between 44.40 to 90.70 µM TE/100 g DW, depending on the storage time [47].

The potato cultivars with the highest content, grown in Cutuglahua, were: *Yellow Chaucha, Natividad, Yana Shungo* and *Victoria* with a 520.69–789.19 μg of TE/g FW range. In Pujilí, the cultivars *Red chaucha, Puca shungo, Natividad* and *Yana shungo* showed results with a 465.35–619.82 μg of TE/g FW range. In Pilahuín the potato cultivars with higher AC were *Black coneja, Libertad, Puca shungo* and *Red chaucha* with a 365.27–585.88 μg of TE/g FW range. Nzaramba et al. (2013) [48] reported a study with 15 advanced breeding selections and three potato cultivars grown in Texas (USA) in 2005. They found antioxidant capacity using the ABTS method with values between 713.40–3999.00 μg of TE/g FW and TPC between 13.20 to 106.10 mg GAE/g FW. They reported a correlation between AC and TPC.

### 3.6. Analysis of Correlation

Pearson correlation analysis showed a significant correlation between AA and AC of r = 0.31 (*p* < 0.05), between AA and TPC of *r* = 0.64 (*p* < 0.01), between TCC and TAC of *r* = −0.28 (*p* < 0.05), between TPC and TAC of *r* = 0.42 (*p* < 0.01), between TPC and AC of *r* = 0.64 (*p* < 0.01) and, finally, between TAC and AC of *r* = 0.25 (*p* < 0.05) (Table 3). Similar results were reported by Leo et al. (2008) [49] when studying the antioxidant activity in 4 varieties of early potatoes in Italy. In conclusion, the contents of TPC and AA would be the most important components in the AC expression in potatoes. The selection of genotypes with high contents of these two phytonutrients will indirectly allow the selection of genotypes with a high AC. Albishi et al. (2013) reported the analysis of the contribution of free, esterified and bound phenolic to antioxidant activity evaluated by ABTS, DPPH, FRAP, and oxygen radical absorbance capacity (ORAC) methods of four varieties potatoes from Canada. The correlation between the total phenolic and antioxidant activity was determined using the Pearson correlation test. They found that the total bound phenolic showed a positive and strong relation with the antioxidant activity [43].

### 3.7. Effect of Cultivar, Location, and Their Interaction with Antioxidant Compounds from Potatoes

The combined analysis for AA (Table 4) showed a statistical significance (*p* < 0.01) for the G x L interaction, the genotype factor, and the location. The contribution of the variation of the genotype (46.90%) was greater than the location factor (16.12%) and the G x L interaction (19.80%). The selection of parents with high AA contents shows the possibility to develop improved genotypes with this character. Similar results were reported by Burgos et al. (2009a) [27] on native CIP cultivars and results reported by Love Pavek (2008) for the North American cultivars [50]. Except for the variety of Cutuglahua that did not show significant differences in the contents through the localities, most of the genotypes presented significant differences for the G x L interaction. The AA averages of the genotypes grown in Cutuglahua were higher compared to the other sites. In Cutuglahua, there were optimal conditions for the development of the crop.

The combined ANOVA for TCC (Table 4) established statistical differences (*p* < 0.01) for the G x L (genotype and location) interaction. The factor that contributed most to the variation was the genotype, 73.10%, followed by the location 3.70%, having a significant interaction between factors explaining the 21.50% of the total variance. These results are in accordance with those reported by Cuesta (2013) [41], in native potato cultivars and by Liu et al. (2004) [24] in diploid varieties and by Nesterenko and Sink (2003) [51], in tetraploid breeding clones. Genotypes such as *Red chaucha* and *Yellow chaucha* are good alternatives to use in breeding for the development of new varieties with high TCC contents. Despite the G x L interaction for most of the genotypes, the *Red chaucha* and *Yellow chaucha* varieties showed the highest TCC concentrations in the three locations. There was a tendency towards higher TCC in the location of Cutuglahua and lower TCC in Pujilí.

The combined ANOVA for TPC established statistical significance (*p* < 0.01) for the G x L interaction, for the genotype factor and for location. The greatest contribution to total variation for TPC was the genotype (45.20%) compared to the location (11.60%) and the G x L interaction (30.50%). Similar results were reported by Cuesta (2013) [41], when evaluating the TPC content in native Ecuadorian varieties. In addition, several authors have reported the significant effect of the genotype factor on the variation of TPC when they studied European varieties. Andre et al. (2007) [11] evaluated the effect G x L in 13 Andean varieties of the CIP. The average TPC contents in the location of Cutuglahua were greater than the other two sites, as they were managed under the conditions of the Experimental Station of the INIAP, in comparison to the other two locations that were sown under local farmer conditions.

The TAC combined analysis (Table 4) established statistical significance (*p* < 0.01) for the G x L interaction, the genotype factor, and the location. The factor that contributed the most to the TAC variation was the genotype (80.50%) compared to the location (0.20%) and the interaction G x L (15.10%). Similar results were reported by Brown (2008) who studied the variability of potato phytonutrients content in relation to the location [44].

Pujilí presented a lower average TAC content compared to the other two locations, suggesting that higher altitudes can reduce TAC. This result is different to the one proposed by Brown (2008) [44] who observed higher TAC at higher altitudes. However, in our study, potato genotypes were evaluated at higher altitudes (3058–3489 masl) compared to the study by Brown (2008) [44] in which the maximum altitude was 1200 masl, which probably affected the TAC. INIAP has described that the altitude of the crop affects the color of the potato skin. This has been observed in *Fripapa* variety with normal pink skin shifting to yellow when being cultivated at altitudes >3200 masl. Varieties with pigmented flesh also change their color due to the altitude of the crop.

The genotypes pigmented with the highest TAC are important sources of anthocyanins, natural pigments widely distributed in fruits and vegetables with positive effects on human health. These pigments can be used for the development of functional or nutraceutical foods in the food industry. The cost of potato production is relatively low compared to other horticultural crops [37,52].

The combined analysis for AC (Table 4), established significant differences *p* < 0.01 for the G x L interaction and the genotype and locality factors. The greater variation was because of the G x L interaction (41.20%) and the effect of the genotype (40.50%) in comparison with the effect of the locality (11.90%). Similar results were reported by Redivari et al. (2007) [45] when studying the effect of genotype, location, and year on the antioxidant capacity of 25 potato varieties. The highest AC was reported for Cutuglahua and the lowest for Pilahuín. These results were probably related to the concentrations of AA, TPC, and TAC described in these locations.

## 4. Conclusions

The potato cultivars tested showed a great variation of their phytochemical content. These differences were produced by the growing environment and genotype. The greater variation of phytochemical component is related to the genotype. Through genetic improvement, the genotypes with the highest content of biocompounds, can be used as progenitors. Cultivars with higher contents of these secondary metabolites and antioxidant capacity can be developed. The varieties *Yana shungo, Puca shungo* and *Black coneja* with colored skin and flesh and with the highest contents of TAC, TPC, and AC represent promising sources for the development of improved varieties with high contents of these compounds. The varieties *Yellow chaucha* and *Red chaucha* with intense yellow flesh presented the highest TAC contents. These varieties constitute promising genotypes for use in breeding for the development of varieties with high contents of this antioxidants. The variety *Libertad* could be used as a parent to improve the AA content. TPC and AA contents are important components in the expression of the AC in potatoes. The selection of genotypes with high contents of these two phytonutrients will indirectly allow the selection of genotypes with a high AC.

## Figures and Tables

**Table 1:** Content of vitamin C (AA), total carotenoid content (TCC), total polyphenol content (TPC), and total anthocyanins content (TAC) in 11 potato cultivars grown in three locations of the Ecuadorian highlands.

	AA					TCC				TPC				TAC		
	(mg AA/100 g FW) ± SD					(µg of β-carotene/g FW) ± SD				(g GAE/kg DW) ± SD				(µg cy-3-glu/100 g FW) ± SD		
Cultivar	Cutuglahua	Pujilí	Pilahuín	*p*	Cutuglahua	Pujilí	Pilahuín	*p*	Cutuglahua	Pujilí	Pilahuín	*p*	Cutuglahua	Pujilí	Pilahuín	*p*
*B. coneja*	29.80 ± 5.31 ^b^	13.38 ± 0.71 ^de^	15.01 ± 2.07 ^de^	**	173.81 ± 1.16 ^d^	86.58 ± 8.66 ^ef^	208.32 ± 3.82 ^cd^	**	1.10 ± 0.11 ^bc^	0.75 ± 0.01 ^cdef^	0.76 ± 0.06 ^cde^	**	27.75 ± 1.51 ^b^	14.01 ± 2.57 ^cd^	36.70 ± 1.77 ^ab^	**
*Y. chaucha*	20.70 ± 4.47 ^ab^	21.46 ± 1.83 ^bcd^	25.05 ± 2.99 ^ab^	**	636.41 ± 3.91 ^b^	341.20 ± 3.59 ^a^	347.71 ± 2.91 ^b^	**	0.93 ± 0.19 ^c^	1.33 ± 0.25 ^ab^	1.44 ± 0.20 ^a^	**	7.13 ± 0.25 ^de^	4.00 ± 1.91 ^e^	5.69 ± 0.47 ^f^	**
*R. chaucha*	25.50 ± 5.34 ^ab^	31.84 ± 2.17 ^ab^	22.63 ± 4.44 ^abc^	**	1043.51 ± 2.38 ^a^	423.46 ± 2.81 ^a^	511.56 ± 2.60 ^a^	**	1.06 ± 0.12 ^bc^	1.18 ± 0.14 ^abc^	1.29 ± 0.23 ^ab^	**	15.01 ± 0.19 ^bc^	14.76 ± 0.33 ^cd^	15.20 ± 0.43 ^cd^	**
*Fripapa*	23.50 ± 2.21 ^ab^	17.99 ± 0.51 ^cde^	13.06 ± 2.93 ^e^	**	87.74 ± 3.78 ^f^	78.47 ± 6.29 ^f^	207.63 ± 1.42 ^d^	**	0.98 ± 0.17 ^bc^	0.53 ± 0.06 ^ef^	0.65 ± 0.14 ^de^	**	29.65 ± 3.54 ^b^	22.34 ± 0.80 ^cd^	15.70 ± 3.26 ^cde^	**
*Libertad*	39.50 ± 0.85 ^a^	33.66 ± 3.73 ^a^	29.07 ± 2.58 ^a^	ns	195.96 ± 9.46 ^d^	163.84 ± 1.84 ^cd^	207.00 ± 2.16 ^cd^	**	0.73 ± 0.08 ^c^	1.16 ± 0.26 ^abc^	0.87 ± 0.44 ^cd^	**	4.73 ± 1.21 ^ef^	2.74 ± 3.83 ^e^	7.25 ± 0.38 ^ef^	**
*Natividad*	30.00 ± 7.92 ^ab^	16.83 ± 6.34 ^cd^	17.44 ± 0.35 ^bcd^	**	167.52 ± 2.27 ^d^	181.12 ± 1.39 ^bc^	211.78 ± 1.98 ^cd^	**	1.14 ± 0.19 ^bc^	1.40 ± 0.29 ^ab^	1.13 ± 0.08 ^abc^	**	6.02 ± 1.66 ^ef^	12.24 ± 1.63 ^cd^	12.99 ± 0.88 ^cde^	**
*P. shungo*	23.40 ± 1.88 ^ab^	23.56 ± 0.20 ^abc^	22.83 ± 1.58 ^abc^	**	100.64 ± 2.89 ^ef^	49.97 ± 1.42 ^g^	220.75 ± 1.44 ^cd^	**	1.87 ± 0.34 ^ab^	1.77 ± 0.06 ^a^	0.82 ± 0.34 ^cde^	**	107.43 ± 7.38 ^a^	62.21 ± 6.63 ^b^	34.67 ± 9.21 ^ab^	**
*Puña*	20.80 ± 0.29 ^b^	12.67 ± 0.93 ^e^	15.29 ± 2.00 ^de^	**	199.88 ± 1.93 ^d^	121.51 ± 2.89 ^de^	57.79 ± 7.22 ^e^	**	1.17 ± 0.26 ^bc^	0.62 ± 0.02 ^def^	0.60 ± 0.04 ^de^	**	25.07 ± 0.40 ^b^	26.54 ± 0.26 ^bc^	18.82 ± 1.32 ^bc^	**
*Uvilla*	32.40 ± 2.92 ^ab^	15.72 ± 0.60 ^cde^	27.59 ± 0.48 ^ab^	**	354.35 ± 7.64 ^c^	212.39 ± 1.04 ^b^	427.86 ± 2.13 ^b^	**	0.97 ± 0.13 ^bc^	0.41 ± 0.03 ^f^	0.57 ± 0.09 ^e^	**	11.99 ± 0.73 ^cd^	3.46 ± 1.76 ^e^	11.51 ± 2.18 ^cdef^	**
*Victoria*	17.10 ± 4.99 ^b^	17.19 ± 0.15 ^cd^	15.86 ± 0.59 ^bcde^	**	127.95 ± 1.76 ^de^	177.91 ± 4.33 ^bc^	224.13 ± 1.75 ^c^	**	1.43 ± 0.42 ^bc^	0.99 ± 0.03 ^bcd^	0.63 ± 0.08 ^de^	**	3.28 ± 0.76 ^f^	8.77 ± 1.05 ^d^	7.81 ± 1.13 ^def^	**
*Y. shungo*	21.80 ± 6.24 ^ab^	24.38 ± 4.52 ^abc^	15.03 ± 6.69 ^cde^	**	132.03 ± 1.27 ^de^	79.00 ± 1.98 ^f^	206.72 ± 5.77 ^cd^	**	3.25 ± 0.40 ^a^	1.65 ± 0.01 ^ab^	0.90 ± 0.34 ^bcd^	**	54.53 ± 5.50 ^a^	172.53 ± 5.45 ^a^	43.37 ± 4.33 ^a^	**

Values are means ± standard deviation (SD) of three determinations. Mean in the column with different letters (^a^–^g^) indicate significant differences between cultivars for each location according to the Tukey *p* test < 0.01. *B. coneja* (*Black coneja*), *Y. chaucha* (*Yellow chaucha*), *R. chaucha* (*Red chaucha*), *B. Libertad* (*Black libertad*), *P. shungo* (*Puca shungo*) and *Y. shungo* (*Yana shungo*). ** significant at 1.00%. ns (not significant). FW: fresh weight.

**Table 2:** Antioxidant capacity by ABTS method of 11 potato cultivars grown in Ecuador.

	ABTS (µg de TE/g FW)			
Genotype	Cutuglahua	Pujilí	Pilahuín	*p < 0.01*
*B. coneja*	438.10 ± 4.37 ^cd^	203.98 ± 2.10 ^cd^	377.57 ± 3.31 ^ab^	**
*Y. chaucha*	520.69 ± 6.26 ^bc^	331.32 ± 2.39 ^abcd^	300.56 ± 1.16 ^abc^	**
*R. chaucha*	279.63 ± 4.21 ^ef^	619.82 ± 3.76 ^a^	439.76 ± 6.06 ^a^	**
*Fripapa*	422.70 ± 2.62 ^cd^	176.02 ± 2.31 ^d^	213.66 ± 1.42 ^bcd^	**
*Libertad*	228.75 ± 2.55 ^f^	304.14 ± 7.33 ^bcd^	365.27 ± 2.66 ^ab^	**
*Natividad*	653.81 ± 7.34 ^b^	504.77 ± 3.08 ^ab^	326.57 ± 6.35 ^ab^	**
*P. shungo*	423.16 ± 4.87 ^cd^	581.97 ± 7.25 ^a^	585.88 ± 7.12 ^a^	**
*Puña*	415.98 ± 6.25 ^de^	82.95 ± 1.72 ^e^	106.30 ± 1.23 ^d^	**
*Uvilla*	424.03 ± 3.21 ^cd^	36.38 ± 2.15 ^f^	335.06 ± 1.86 ^abc^	**
*Victoria*	789.19 ± 5.95 ^a^	328.46 ± 1.55 ^abc^	134.85 ± 4.12 ^cd^	**
*Y. shungo*	751.76 ± 5.13 ^a^	465.35 ± 4.26 ^ab^	332.41 ± 7.45 ^ab^	**

Values are means ± standard deviation (SD) of three determinations. Mean in the column with different letters (^a^–^f^) indicate significant differences between genotypes for each location according to the Tukey *p* test < 0.05. *B. coneja* (*Black coneja*), *Y. chaucha* (*Yellow chaucha*), *R. chaucha* (*Red chaucha*), *B. Libertad* (*Black libertad*), *P. shungo* (*Puca shungo*) and *Y. shungo* (*Yana shungo*). ** significant at 1.00%. TE: Trolox Equivalent.

**Table 3:** Pearson correlation coefficient (r) between the analytical parameters: content of vitamin C (AA), total carotenoids content (TCC), total polyphenols compounds (TPC), total anthocyanins content (TAC), and antioxidant capacity (AC) of 11 potato cultivars grown in Ecuador.

	AA	TCC	TPC	TAC	AC
Vitamin C (AA)	1.00	0.16	0.23 *	0.07	0.31 **
Carotenoids (TCC)		1.00	–0.06	–0.28 *	–0.03
Polyphenols (TPC)			1.00	0.42 **	0.64 **
Anthocyanins (TAC)				1.00	0.25 *
Antioxidant capacity (AC)					1.00

* Significant correlation at 95.00% probability; ** Significant correlation at 99.00% probability.

**Table 4:** Analysis of the variance (ANOVA) combined and proportion of the variance for the content of vitamin C (AA), total carotenoids content (TCC), total polyphenol content (TPC), total anthocyanins content (TAC), and antioxidant capacity (AC) of potatoes cultivars grown in Ecuador.

		AA		TCC		TPC		TAC		AC	
Origin of Variety	DF	MS	%VT	MS	%VT	MS	%VT	MS	%VT	MS	%VT
Total	98.00	0.28	100.00	0.83	100.00	0.43	100.00	1.74	100.00	8.22	100.00
Repetition	2.00	4.60E-03ns	0.50	2.20E-03 ns	0.10	1.70E-03 ns	0.10	0.03 ns	0.40	0.01 ns	0.10
Genotype G)	10.00	0.09 **	**46.90**	0.59 **	**73.10**	0.16 **	**45.20**	1.54 **	**80.50**	3.33 **	**40.50**
Location (L)	2.00	0.16 **	**16.12**	0.15 **	**3.70**	0.21 **	**11.60**	0.02 **	**0.20**	0.98 **	**11.90**
G x L	20.00	0.02 **	**19.80**	0.09 **	**21.50**	0.05 **	**30.50**	0.14 **	**15.10**	3.39 **	**41.20**
Error	64.00	4.90E-03	16.70	2.20E-03	1.70	0.01	12.70	0.01	3.90	0.51	6.20

MS (Mean Square), % VT (Proportion of the total variance), ns (not significant at 95.00%); ** (Significant at 99.00% probability). DF (degree of freedom).
